# Seed Germination Ecology of Semiparasitic Weed *Pedicularis kansuensis* in Alpine Grasslands

**DOI:** 10.3390/plants11131777

**Published:** 2022-07-05

**Authors:** Jiedong Hu, Kaihui Li, Chengjun Deng, Yanming Gong, Yanyan Liu, Lei Wang

**Affiliations:** 1 CAS Research Center for Ecology and Environment of Central Asia, Xinjiang Institute of Ecology and Geography, Chinese Academy of Sciences, Urumqi 830011, China; hujiedong19@mails.ucas.ac.cn (J.H.); gongym@ms.xjb.ac.cn (Y.G.); liuyany@ms.xjb.ac.cn (Y.L.); 2 University of Chinese Academy of Sciences, Beijing 100049, China; 3 Bayinbuluk Grassland Ecosystem Research Station, Xinjiang Institute of Ecology and Geography, Chinese Academy of Sciences, Bayinbuluk 841314, China; 4 Engineer, Grassland Station of Bayingol Mongolian Autonomous Prefecture of Xinjiang, Korla 841000, China; grassland1998@126.com; 5 State Key Laboratory of Desert and Oasis Ecology, Xinjiang Institute of Ecology and Geography, Chinese Academy of Sciences, Urumqi 830011, China

**Keywords:** cold stratification, light, temperature, osmotic stress, plant growth regulator, seed germination

## Abstract

The semiparasitic weed *Pedicularis kansuensis* Maxim. has rapidly spread in the alpine grasslands of northern China over the past twenty years and has caused serious ecological problems. In order to effectively halt the spread of this weed, a thorough understanding of the dormancy type and the seed-germination ecology of *P. kansuensis* is required. We have conducted a series of experiments to investigate the effects of plant growth regulators (gibberellin (GA_3_) and strigolactone synthesis (GR24)), as well as different abiotic (temperature, light, cold stratification, and drought) and biotic (aqueous extracts of three native dominant plants) factors on the seed-germination characteristics of *P. kansuensis*. The seed-germination percentages ranged from 2% to 62% at all of the temperatures that were examined, with the highest occurring at 25/10 °C. The light conditions did not significantly affect the germination percentage. The seed germination was greatly improved after two to eight weeks of cold stratification. The seed germination decreased dramatically with an increasing polyethylene glycol (PEG-6000) concentration, from 55% to 0%, under 10% and 20% PEG-6000. The seed germination was improved at a proper concentration of GA_3_, GR24, and the aqueous extracts of *Festuca ovina* L., *Stipa purpurea* L., and *Leymus secalinus* (Georgi) Tzvel. Furthermore, in the pot experiment, the seedling emergence of *P. kansuensis* was also improved by the cultivation of these three dominant grasses. These findings indicate that the dormancy type of *P. kansuensis* seeds is non-deep physiological dormancy, and such findings will help in paving the way for the creation of effective weed management strategies, based on a thorough knowledge of germination ecology.

## 1. Introduction

China has the second largest grazing grassland in the world [1]. The stability of the rangeland ecosystems might be weakened due to overgrazing and human activities, which aggravate the invasion of poisonous and harmful plants that can lead to grassland degradation. Xinjiang has 5.7 × 10^7^ ha of natural grassland, which is the third largest steppe in China [2]. About 60% of the grassland in Xinjiang is distributed in the mountainous areas and high basins [3]. The Bayinbuluk alpine grassland is located in the central Tianshan Mountains, with an average elevation of 2500 m and an average annual temperature of −4.8 °C. It has distinct alpine climate characteristics. Gramineae, such as *Festuca ovina* L. and *Stipa purpurea* L., are the main native species in the Bayinbuluk alpine grassland. However, *Pedicularis kansuensis* Maxim. have expanded rapidly in this area since the year 2000. This species is a typical expansive species in China, which has brought great harm to the local animal husbandry [3,4,5].

*Pedicularis kansuensis* is an annual or biennial semiparasitic plant. The flowering period is from June to August and the fruit period is from July to September. The habitat of this species is gravelly ground and grassy slopes in subalpine zones, damp grassy areas along field margins, damp slopes, and valleys. This plant mainly grows in the Xinjiang, Gansu, Qinghai, Sichuan, and Yunnan provinces in China [3,6]. The plant has high adaptability and spreads rapidly in the pasture in the form of cluster distribution over a short period of time, showing a strong invasion ability [3,4]. The damaged areas of *P. kansuensis* have reached 4.1 × 10^4^ ha in the Bayinbuluk alpine grassland, Xinjiang, China, and the spread rate is about 3.3 × 10^3^ ha per year [7,8]. *P. kansuensis* has a strong inhibitory effect on grass growth, is unpalatable to livestock, and hence impacts the development of the local animal husbandry [3].

The expansion of most plants depends on the seed dispersal and the successful establishment of new populations. Thus, seed germination is a crucial stage in the plant’s life cycle [9,10]. Some studies indicate that the expansion of poisonous grass is positively correlated with the ability of seed germination [11,12,13]. Seed germination, under natural conditions, is generally affected by temperature, light, soil moisture, salinity, plant allelopathic substances, etc., [14,15,16,17]. During seed germination, the appropriate temperature is required for the decomposition of the storage substances and the efficient operation of the enzymes that are related to the synthesis of new substances [18]. Light plays a role in signal transmission during germination and can either positively or negatively regulate seed germination [19]. Plant hormones, such as gibberellin and strigolactone, can improve the embryo development and the seed germination of weeds [20,21,22].

*Pedicularis kansuensis* has previously been studied for its potential geographic distribution under climate change [23], endophytic association [24], allelochemical inhibitory activity [25], and germination [26,27]. The germination percentage is significantly increased by the increase in temperature [3,27]. However, there have been few thorough investigations on the germination responses to various abiotic and biotic variables. Furthermore, no study has been undertaken to determine the dormancy type of *P. kansuensis* seeds. This study looks into the seed-germination ecology of the semiparasitic plant *P. kansuensis*. The main goal of this study was to assess the dormancy type and the requirements for the seed germination of this species in the Bayinbuluk alpine grassland, which is a fragile ecosystem that is particularly threatened by global climate change and the expansion of noxious grasses. We hypothesized that (1) the seeds of this species have non-deep physiological dormancy, as do most weeds; (2) light and cold stratification could increase their germination; (3) the native dominant plants are able to increase their seed germination and seedling emergence. In order to test these hypotheses, we collected *P. kansuensis* seeds and used laboratory experiments to test the germination responses to temperature, light, cold stratification, drought stress, plant hormones, and the aqueous extracts of three native dominant plants. Finally, we assessed how the seedling emergence of this plant responded to the transplant of these native dominant plants.

## 2. Results

### 2.1. Seed Morphology, Mass, and Imbibition

**Figure 1:**
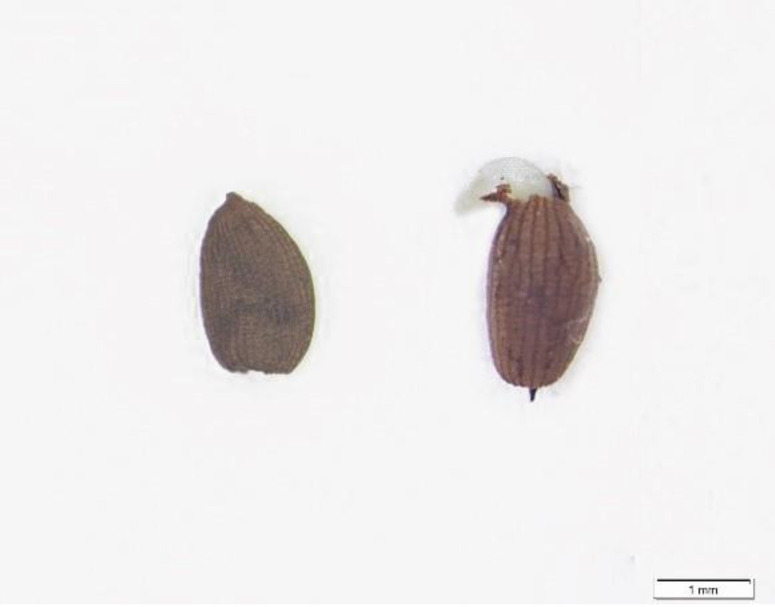
Electron microscope pictures of seeds before and after germination.

The seed coat color of *P. kansuensis* is dark-brown and the shape is oval (Figure 1). The 1000-seed weight was 861 ± 14 mg. The average length and width of seeds were 2137 ± 213 µm and 1051 ± 110 µm, respectively. An imbibition test showed that the seed weight increased by 7.7%, 23.1%, 36.1%, 69.2%, and 86.1% after imbibing water for 1 h, 5 h, 10 h, 30 h, and 70 h, respectively.

### 2.2. Effect of Temperature and Light on Seed Germination

**Figure 2:**
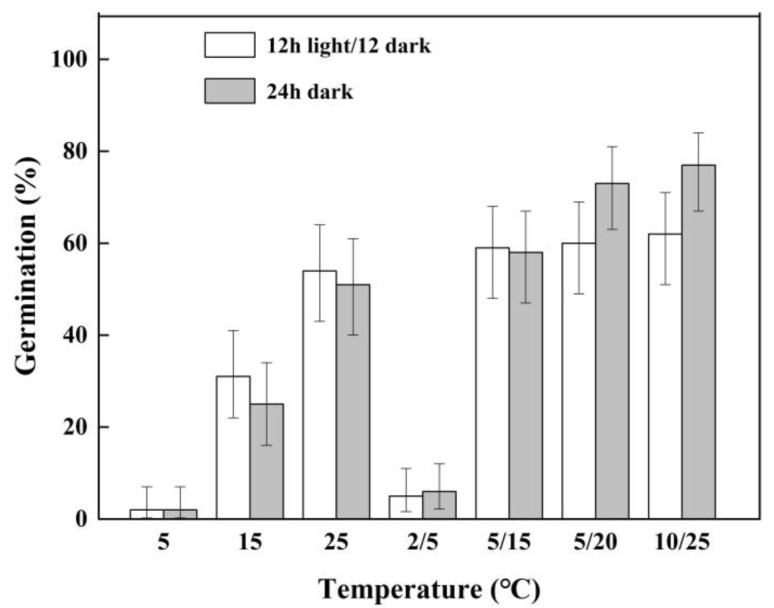
Germination percentage (±95% binomial confidence intervals) of *P. kansuensis* seeds incubated under different temperature and light conditions.

The germination of *P. kansuensis* seeds was significantly affected by temperature (P < 0.05) but by not light (P > 0.05) or the interaction between temperature and light (P > 0.05). The germination percentages of the seeds gradually increased as the temperature approached 25 °C, which reached the maximum (77%) at a temperature of 10/25 °C, with 24 h darkness, and the lowest (2%) at a temperature of 5 °C. The percentages of seed germination under fluctuating temperatures of 2/5, 5/15, and 10/25 °C were higher than that under the corresponding constant temperature of 5, 15, and 25 °C, respectively (Figure 2).

### 2.3. Effect of Cold Stratification on Seed Germination

**Figure 3:**
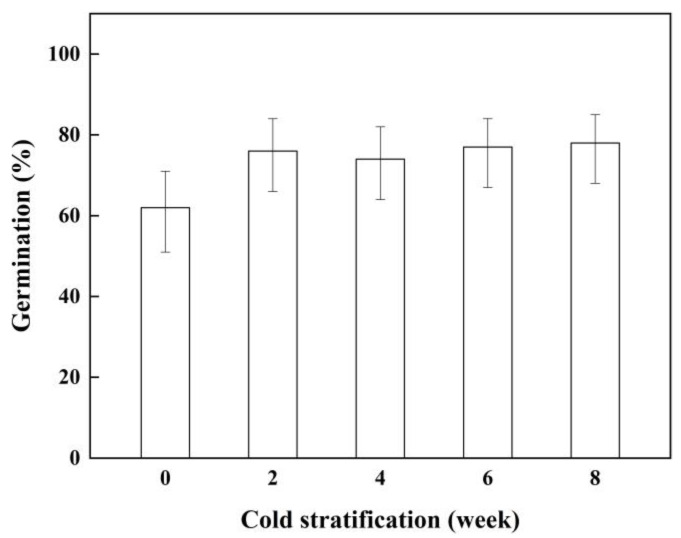
Germination percentage (±95% binomial confidence intervals) of *P. kansuensis* seeds incubated with different cold stratification treatments in 12 h light/12 h dark at 10/25 °C.

The germination percentages of the seeds increased dramatically after treatment with two, four, six, and eight weeks of cold stratification (P < 0.05). The maximum germination percentage of the seeds was 78% at eight weeks. However, the germination percentage of the seeds reached 76% at two weeks, which was close to the maximum value. The different cold stratification periods had similar effects on seed germination (Figure 3).

### 2.4. Effect of Drought Stress on Seed Germination

**Figure 4:**
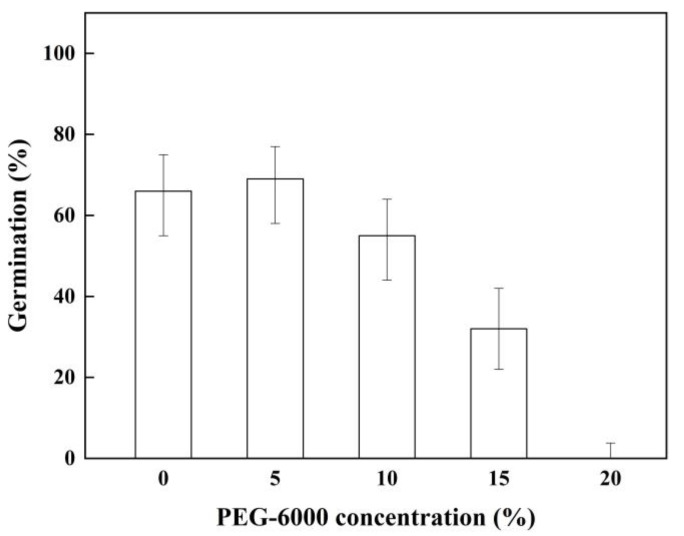
Germination percentage (±95% binomial confidence intervals) of *P. kansuensis* seeds treated with different PEG-6000 concentrations in 12 h light/12 h dark at 10/25 °C.

The seed germination was significantly affected by drought stress at higher concentrations of PEG-6000. The seed-germination percentages decreased with the increase in concentration. The germination percentage of the seeds decreased by 16.7% and 51.5% at the PEG-6000 concentrations of 10% and 15%, respectively, compared with that at a 0% concentration. Moreover, it was completely inhibited at a PEG-6000 concentration of 20% (Figure 4).

### 2.5. Effect of Plant Hormones on Seed Germination

**Figure 5:**
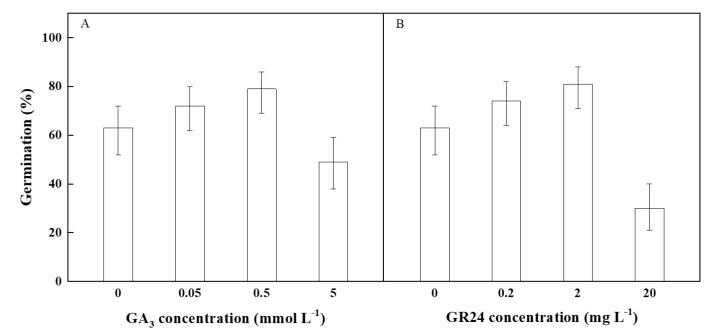
Germination percentage (±95% binomial confidence intervals) of *P. kansuensis* seeds treated with different concentrations of (**A**) GA_3_ and (**B**) GR24 in 12 h light/12 h dark at 10/25 °C.

Both types of the plant hormones significantly affected the seed germination percentage. With the increase in GA_3_ and GR24 concentrations, the seed germination percentages increased at low concentration, reached the highest value at the medium concentration, and then decreased at the highest concentration. The highest percentage of seed germination was 79% at 0.5 mmol L^−1^ of GA_3_. However, the seed-germination percentage was the lowest at the maximum concentration with 5 mmol L^−1^ of GA_3_ (Figure 5A). The percentage of seed germination reached the maximum (81%) and minimum (30%) at the GR24 concentrations of 2 mg L^−1^ and 20 mg L^−1^, respectively (Figure 5B).

### 2.6. Effect of Aqueous Extracts of Native Dominant Plants on Seed Germination

**Figure 6:**
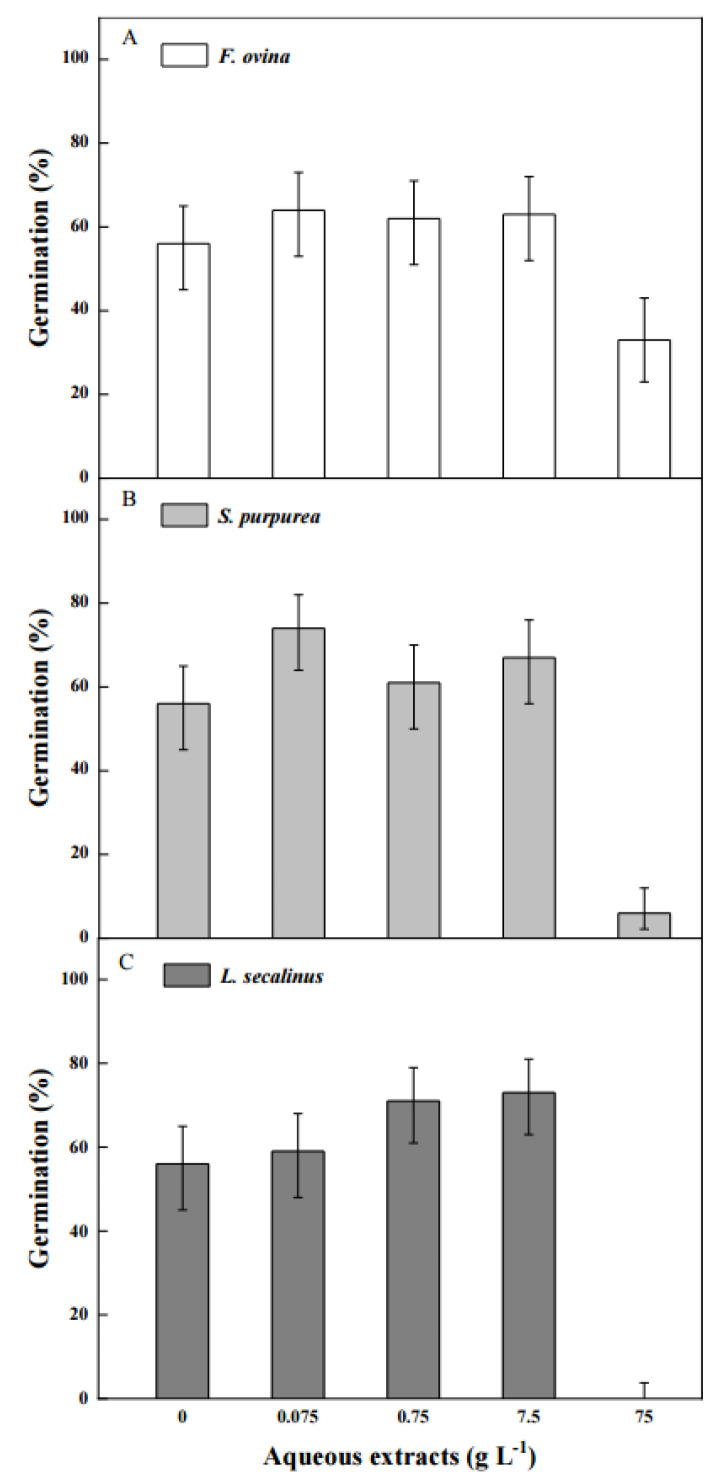
Germination percentage (±95% binomial confidence intervals) of *P. kansuensis* seeds treated with different contents of aqueous extracts of three native dominant plants ((**A**), *F. ovina*; (**B**), *S. purpurea*; (**C**), *L. secalinus*) in 12 h light/12 h dark at 10/25 °C.

The germination of the *P. kansuensis* seeds was significantly affected by the aqueous extracts of the native dominant perennial grasses. With the increase in the aqueous extract concentrations of the native grasses, the seed germination percentages first increased and then decreased (Figure 6). The seed germination of *P. kansuensis* was almost completely inhibited at the 75 g L^−1^ concentration of the *S. purpurea* and *L. secalinus* extracts and the germination percentages were 6% and 0%, respectively. In addition, the inhibition effect of the aqueous extracts of *F. ovina* was less than that of *S. purpurea* and *L. secalinus*.

### 2.7. Effect of Transplantation of Native Dominant Plants on Seedling Emergence

**Figure 7:**
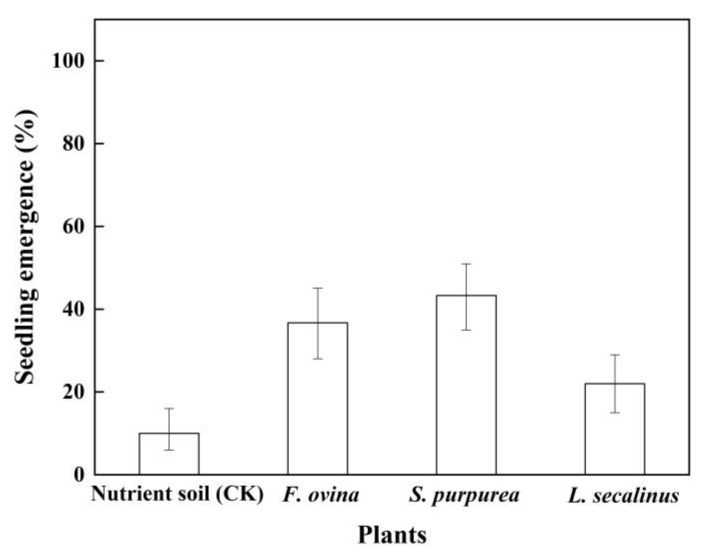
Seedling emergence (±95% binomial confidence intervals) of *P. kansuensis* treated with transplantation of different native dominant plants.

The seedling emergence of *P. kansuensis* was significantly increased by the transplantation of the three native dominant grasses in a pot experiment (Figure 7). The seedling-emergence percentage of *P*. *kansuensis* reached the highest value (43.3%) in the pot environment of *S. purpurea*, but it reached the lowest value (10%) in the control group. *F. ovina* and *S. purpurea* had the highest promoting effect on the seedling emergence, followed by *L. secalinus*.

## 3. Discussion

Although the basic germination characteristics of *P. kansuensis* have been studied previously [27], these data are the first that document the dormancy type and the effects of the native dominant plants on the germination and seedling emergence. In addition, our findings suggest that the dormancy type of this plant is non-deep physiological dormancy (PD). Cold stratification, but not light, could significantly increase the seed-germination performance. The results indicate that, not only was the seed germination increased by a moderate concentration of the aqueous extracts of native dominant plants, but the seedling emergence was also promoted by the existence of these plants.

An imbibition test indicated that, although the rate of water absorption of *P. kansuensis* seeds was quite low, the seeds were indeed water-permeable. In the dormancy classification system of Baskin and Baskin (2004, 2014) [28,29], seeds with PD, morphological dormancy (MD), and morphophysiological dormancy (MPD) have a water-permeable seed coat. In addition, the seeds of *P. kansuensis* have fully developed embryos. Thus, we may conclude that *P. kansuensis* seeds exhibit PD. PD is divided into three levels (non-deep, intermediate, and deep) by Baskin and Baskin (2004, 2014) [28,29]. We found that two–eight weeks of cold stratification could significantly increase the germination of *P. kansuensis* seeds and GA_3_ also promoted germination. Based on these characteristics, we can conclude that seeds of *P. kansuensis* have non-deep PD.

Temperature is the main limiting factor in regulating germination timing in the field. The temperature not only affects the induction and breaking of non-deep PD, but also influences the seed-germination percentage and velocity [29,30]. Our results indicate that several weeks of cold stratification can effectively promote the germination of *P. kansuensis* seeds. We can infer that the seed dormancy of this plant is released under low temperatures from late autumn to early spring, and then non-dormant seeds can germinate after the snowmelt in the middle of April. The temperature is a good indicator of season changes and is thus implicated in determining the timing of germination. With the increase in temperature, the seed-germination percentage of this plant gradually increased and reached the highest germination at 10/25 °C, which was closely linked to the observed timing of emergence in the field. The germination is reduced at constant temperatures and increased by the temperature alternations in *P. kansuensis*. This phenomenon is similar to the response of the seed germination of *Pistacia vera* L. and *Eleusine indica* [31,32]. Diurnal temperature alternations decline with soil depth and, thus, also play a crucial role in regulating the location of germination.

In the field, seed germination, which may take several days or weeks, will probably encounter periods of drought. Crucial aspects in the germination process are the soil water potential and the length of the hydration period [33,34]. When the soil water potential is suitable (<15% PEG) for *P. kansuensis*, the seeds begin to germinate after absorbing enough water. However, when the soil water potential is too high and the length of the hydration period is short, the germination may not proceed to the critical physiological point and the seeds can be dehydrated in the field [35]. *P. kansuensis* seeds cannot germinate at 20% PEG, which can be seen as medium drought tolerance. The effects of the drought levels and the length of the dry period on this plant should receive more attention in future research because the viability and the germination velocity of many species are affected by these factors [36,37,38].

The seed germination of *P. kansuensis* was increased by the low to medium concentrations of the aqueous extracts of the native dominant grasses, *F. ovina*, *S. purpurea,* and *L. secalinus*. Furthermore, the seedling emergence of *P. kansuensis* was promoted by the existence of *F. ovina*, *S. purpurea,* and *L. secalinus*. Although *P. kansuensis* is hemiparasitic, our results indicate that the germination of the seeds is completely independent of its host. However, the proper concentrations of GA_3_ and GR24 can stimulate the germination of this plant, and the chemical signal from their host can increase their chances of emerging from the soil. Generally, the suicidal germination of the seeds in order to reduce the soil seed bank and inhibiting germination are the two main strategies for controlling parasitic and semiparasitic weeds [39,40,41]. Thus, GA_3_, GR24, and the aqueous extracts of the native dominant plants may increase the effectiveness of suicidal germination for *P. kansuensis* control.

## 4. Materials and Methods

### 4.1. Seed Collection

Freshly matured seeds were collected from more than 50 *P. kansuensis* individuals in September 2020 at the Bayinbuluk Grassland Station of the Xinjiang Institute of Ecology and Geography, Chinese Academy of Sciences (42°53′05.56′′ N, 83°42′32.43′′ E). After naturally drying in the laboratory, the seeds were stored at room temperature (20 ± 3 °C) until being used in this experiment.

### 4.2. Seed Morphology, Mass, and Imbibition

The length and width of 20 seeds were measured by a microscope using Olympus cellSens software. Then, 4 groups of 1000 seeds were weighed using an electronic analytical balance (Sartorius BP 221 S, Sartorius, Göttingen, Germany).

An imbibition test was conducted at room temperature. The dry mass of each group of 25 seeds was determined (time 0), and the seeds were then placed in 5 cm diameter petri dishes on filter paper moistened with distilled water. After 1 h, the seeds were removed from the petri dishes, blotted dry with filter paper, reweighed, and were returned to the petri dishes. Seed mass was measured again after 5, 10, 30, and 70 h of water absorption. The relative increase in the fresh weight (Wr) of the seeds was calculated as Wr = ((Wf − Wi)/Wi) × 100, where Wi is the initial seed weight and Wf is the weight after a certain time [28].

### 4.3. General Seed-Germination Test

The germination experiment of *P. kansuensis* was carried out in November 2020 and the cold stratification experiment was conducted from November 2020 to April 2021. There were 4 replicates for each treatment and 25 seeds for each petri dish. The seeds were placed in 5 cm diameter petri dishes on 2 layers of filter paper soaked with 2.5 mL of distilled water or test solution. The petri dishes were sealed with parafilm. All experiments were carried out in an incubator at 10/25 °C and the photosynthetic photon flux density was about 100 µmol m^−2^ s^−1^. The radicle ≥1 mm was the criterion for seed germination. For 20 days, the number of germinated seeds in light was counted every 2 days. Following the germination test, the non-germinated seeds were examined under a dissecting microscope. A firm and white embryo meant that the seed was viable, while a soft and grey embryo indicated the seed was non-viable.

### 4.4. Effect of Temperature and Light on Seed Germination

Seeds were germinated at three constant temperatures (5, 15, and 25 °C) and four fluctuating temperatures (2/5, 5/15, 5/20, and 10/25 °C). The light condition was a 12 h light/12 h dark photoperiod. For the continuous darkness treatment, petri dishes were wrapped with two layers of aluminum foil. The number of germinated seeds was counted only after 20 days of incubation. Other experimental conditions were the same as those described above.

### 4.5. Effect of Cold Stratification on Seed Germination

About 1000 seeds were placed on 2 layers of filter paper in 9 cm diameter petri dishes. Then, the seeds were covered with two layers of filter paper and 10 mL of distilled water was added. Petri dishes were sealed with parafilm and then wrapped with two layers of aluminum foil. Then, 4 petri dishes were placed at 2 °C for 2, 4, 6, or 8 weeks. After cold stratification, the germination experiment was carried out in light, according to the general seed-germination test.

### 4.6. Effect of Drought Stress on Seed Germination

Polyethylene glycol (PEG-6000) was used to simulate drought stress; 4 replicates of 25 seeds were incubated in 0%, 5%, 10%, 15%, and 20% PEG-6000 solutions.

### 4.7. Effect of Plant Hormones on Seed Germination

The treatment concentrations of gibberellin (GA_3_) were set as 0.05 mmol L^−1^, 0.5 mmol L^−1^, and 5 mmol L^−1^, and the concentrations of strigolactone synthesis (GR24) were set as 0.02 mg L^−1^, 0.2 mg L^−1^, and 2 mg L^−1^. The control was treated with distilled water.

### 4.8. Effect of Aqueous Extracts of Native Dominant Plants on Seed Germination

Leaves of three native dominant plants (*Festuca ovina* L., *Stipa purpurea* L., and *Leymus secalinus* (Georgi) Tzvel.) were collected from Bayinbuluk Grassland Station in August 2021. Then, the leaves were dried for 48 h at 65 °C, were ground into powder by a mill, and were passed through a 0.18 mm sieve. The extraction process was performed by using an aqueous extract.

The dried powder (75 g) of the different species was put into different beakers, treated with 900 mL of distilled water, and was extracted at room temperature for 48 h. Then, the mixture was filtered through a double-layer gauze, transferred to a volumetric flask, and set to 1000 mL with distilled water. Subsequently, dilutions were performed to achieve the appropriate final concentrations of 0.075 g L^−1^, 0.75 g L^−1^, 7.5 g L^−1^, and 75 g L^−1^. Germination control was treated with distilled water. The other experimental conditions were the same as those described above.

### 4.9. Transplantation of Native Dominant Plants on Seedling Emergence

*F. ovina*, *S. purpurea,* and *L. secalinus* were transplanted into ten pots with a caliber of 16 cm, a bottom diameter of 12.5 cm, and a height of 17.5 cm, respectively, on 10 July 2021. Each plant coverage was about 50% in a pot with 2.5 L nutrient soil. At the same time, 15 plump seeds were sown near the plant root at 0.5 cm in each pot, and the burial depth was 1 cm. The control group was also set up with 10 pots of 2.5 L nutrient soil and had only 15 seeds in each pot. All pots were put in the field at Bayinbuluk Grassland Station. Every day, 70 mL of well water was added to each pot. After 45 days, the number of emerged seedlings was counted.

### 4.10. Statistical Analyses

Germination percentages and seedling emergence under different experimental procedures were analyzed by generalized linear models with a binomial error structure and logit link function. Germination conditions (temperatures and light conditions) and treatments (cold stratification, PEG-6000 concentration, GA_3_ concentration, GR24 concentration, content of aqueous extract of three native dominant plants, and transplantation of three native dominant plants) were considered as fixed factors. Models were performed, taking into account the possible overdispersion of data and the significance level, which was set at 0.05. All statistical analyses were performed using IBM SPSS Statistics 19.

## 5. Conclusions

In conclusion, our results showed that mature seeds of *P. kansuensis* have non-deep PD. This dormancy was partly broken by two to eight weeks of cold stratification. The germination results indicate that the seeds of *P. kansuensis* have a higher germination percentage under fluctuating temperatures than under constant temperatures. However, the seed germination was found to be insensitive to light conditions. The seeds of *P. kansuensis* were moderately tolerant to water stress, with a germination percentage of 32% at a concentration of 15% PEG-6000. Furthermore, the native dominant plants promote the seed germination and the seedling emergence of this weed. Using GA_3_, GR24, and the aqueous extracts of the native dominant plants as natural herbicides, inducing the suicidal germination of *P. kansuensis* seeds might be an option for invasion control. Thus, this study provides thorough information on the ecological requirements for the germination of the semiparasitic weed *P. kansuensis*, which may facilitate the development of effective control measures.

## Data Availability

All data generated during this study are included in this article.
